# Retraction Note: Bridging the gap: robotic applications in cerebral aneurysms neurointerventions - a systematic review

**DOI:** 10.1007/s10143-026-04251-8

**Published:** 2026-03-26

**Authors:** Paweł Marek Łajczak, Bartłomiej Jurek, Kamil Jóźwik, Zbigniew Nawrat

**Affiliations:** 1 Department of Biophysics, Faculty of Medical Sciences in Zabrze, Medical University of Silesia in Katowice, Jordana 18, Zabrze, 40-043 Poland; 2 Foundation of Cardiac Surgery Development, Zabrze, 41-808 Poland

**Retraction Note: Neurosurgical Review (2024) 47:150**

10.1007/s10143-024-02400-5

The Editor-in-Chief has retracted this article. An investigation by the Publisher found that a number of articles, including this one, were submitted over a short space of time and show strong indications that the text was generated by a large language model (LLM) without proper disclosure by the authors. These articles are, therefore, in breach of the Journal's editorial policy and are being retracted.

Paweł Łajczak on behalf of all authors disagrees with the retraction.

